# Associations between digital literacy, health literacy, and digital health behaviors among rural residents: evidence from Zhejiang, China

**DOI:** 10.1186/s12939-024-02150-2

**Published:** 2024-04-09

**Authors:** Hao Ji, Junqiang Dong, Weiguang Pan, Yingying Yu

**Affiliations:** 1 Zhejiang A&F University, College of Economics and Management, Hangzhou, People’s Republic of China; 2 Hangzhou Medical College, Center for Medical Intelligence and Health Policy Research, Hangzhou, People’s Republic of China; 3 Zhejiang A&F University, Mental Health Education Center, Hangzhou, People’s Republic of China; 4 Research Academy for Rural Revitalization of Zhejiang Province, Zhejiang A & F University, Hangzhou, People’s Republic of China

**Keywords:** Digital literacy, Health literacy, Digital health behaviors, Digital health inequality, Digital health divide, Rural residents

## Abstract

**Objective:**

Within the digital society, the limited proficiency in digital health behaviors among rural residents has emerged as a significant factor intensifying health disparities between urban and rural areas. Addressing this issue, enhancing the digital literacy and health literacy of rural residents stands out as a crucial strategy. This study aims to investigate the relationship between digital literacy, health literacy, and the digital health behaviors of rural residents.

**Methods:**

Initially, we developed measurement instruments aimed at assessing the levels of digital literacy and health literacy among rural residents. Subsequently, leveraging micro survey data, we conducted assessments on the digital literacy and health literacy of 968 residents in five administrative villages in Zhejiang Province, China. Building upon this foundation, we employed Probit and Poisson models to empirically scrutinize the influence of digital literacy, health literacy, and their interaction on the manifestation of digital health behaviors within the rural population. This analysis was conducted from a dual perspective, evaluating the participation of digital health behaviors among rural residents and the diversity to which they participate in such behaviors.

**Results:**

Digital literacy exhibited a notably positive influence on both the participation and diversity of digital health behaviors among rural residents. While health literacy did not emerge as a predictor for the occurrence of digital health behavior, it exerted a substantial positive impact on the diversity of digital health behaviors in the rural population. There were significant interaction effects between digital literacy and health literacy concerning the participation and diversity of digital health behaviors among rural residents. These findings remained robust even after implementing the instrumental variable method to address endogeneity issues. Furthermore, the outcomes of robust analysis and heterogeneity analysis further fortify the steadfastness of the aforementioned conclusions.

**Conclusion:**

The findings suggest that policymakers should implement targeted measures aimed at enhancing digital literacy and health literacy among rural residents. This approach is crucial for improving rural residents' access to digital health services, thereby mitigating urban–rural health inequality.

**Supplementary Information:**

The online version contains supplementary material available at 10.1186/s12939-024-02150-2.

## Introduction

In the era of Web 3.0, a new wave of information technologies, including the Internet, big data, cloud computing, the Internet of Things, artificial intelligence, and blockchain, is rapidly evolving. These technologies are significantly optimizing, reshaping, and even transforming traditional service models. Within the realm of public health, the value of digital health technologies is increasingly pronounced, presenting a novel opportunity to enhance the well-being of humanity [66]. For instance, amid the global public health crisis posed by the COVID-19 pandemic, medical service models grounded in digital health technologies—such as remote appointment services, online consultations and examinations, remote imaging diagnostics, and digital pharmaceutical sales—have been widely embraced by numerous countries. These measures aim to mitigate the risk of cross-infection among patients, alleviate the strain on medical resources, and augment the overall efficiency of healthcare services [46]. In the post-pandemic phase, the market for digital health technologies is witnessing a surge in rapid growth. According to the "2023–2027 Global Digital Medical Industry Economic Development Blue Book," the global digital health market surpassed $211 billion in 2022. Projections indicate a consistent annual compound growth rate of 18.6% from 2023 to 2030, culminating in a market size of $809.2 billion. The global trajectory towards digitization in health and healthcare is unmistakable.

Digital health technology is a pivotal complement to traditional healthcare resources that leverages emerging information and communication technology (ICT) to address health-related issues [48]. Distinct from traditional healthcare service models, digital health technology transcends temporal and spatial constraints, offering notable advantages in efficiency, information accessibility, diverse scenarios, and resource utilization, thereby extending the scope of medical services [8]. Recognizing the potential, the World Health Organization asserts that enhancing health services through digital health technology contributes significantly to improving the health and well-being of vulnerable populations [93]. Some scholars even characterize digital health technology as a "super-determinant of social health" [74, 88]. However, an accumulating body of evidence indicates that equitable access to digital health technology is not assured, with significant spatial disparities evident, particularly between urban and rural areas. Residents in remote rural areas encounter substantial barriers to the use of digital health technology [91] and exhibit less positive engagement in digital health behaviors [22, 29]. A survey conducted by Hong et al. [33] found that urban residents in China were approximately twice as likely to engage in digital health behaviors compared to their rural counterparts. The 52nd China Internet Development Status Statistical Report released by the China Internet Network Information Center (CNNIC) in June 2023 reveals that the internet medical coverage rate in rural areas of China was only 22.8% [15]. A recent empirical study in China indicates that rural residents possess weaker capabilities in searching, acquiring, understanding, and utilizing online health information, displaying a lower willingness to participate in digital health behaviors [19]. On a global scale, there exists pervasive inequality in the utilization of digital health services between urban and rural areas. For example, rural residents in Australia demonstrate a lower willingness to use digital health resources [39, 61]. The Healthy People 2030 report reveals that rural residents in the United States have a diminished ability to access online health information compared to their urban counterparts [35]. Studies from India and Italy also indicate that rural residents experience fewer scenarios of digital health use compared to their urban counterparts [66, 85].

A pertinent question arises: why have digital health services, theoretically laden with considerable advantages, not progressed as anticipated in rural areas? Addressing this query becomes a pivotal task, necessitating an in-depth examination of the inhibiting factors that impact the engagement of rural residents in digital health behaviors. Such an inquiry is indispensable for augmenting the capacity of rural residents to access digital health services and, consequently, ameliorating their overall health. Failure to undertake this critical investigation may perpetuate the threat of health inequality between urban and rural areas, a concern highlighted by previous studies [37, 54].

Recent studies underscore the critical role of digital health literacy, also known as eHealth literacy, as a determining factor in user engagement with digital health technologies [13]. Coined by Norman et al. in 2006, digital health literacy is defined as the proficiency to seek, find, understand, evaluate, transform health information, and apply acquired knowledge to address health issues from electronic sources. This comprehensive construct comprises six dimensions: traditional literacy, health literacy, information literacy, scientific literacy, media literacy, and computer literacy [57, 58]. The latest evidence reveals an uneven distribution of digital health literacy within the population, suggesting that not everyone possesses the opportunity and capability to fully leverage the benefits of digital health technologies. This imbalance contributes to the emergence of a digital health divide [73]. The digital health divide signifies the gap between individuals who can access and utilize health information technologies and those who cannot [31]. Although the divide has somewhat alleviated due to the widespread use of the internet and the proliferation of smartphones across populations and regions, digital connectivity in low-income groups and remote areas still lags [6, 64]. Consequently, rural residents emerge as a significant demographic affected by the digital divide, particularly within the domain of digital health and healthcare [90].

Through a comprehensive literature review, it is evident that the existing academic research in the field of digital health has predominantly focused on theoretical interpretations of user digital health literacy [39, 56, 57], its measurement levels [34, 53, 57], and determinants of influencing factors [30]. However, there has been relatively less exploration of the relationship between user digital health literacy and digital health behaviors, which often holds crucial and valuable information determining users' engagement in digital health behavior participation. Recent studies suggest that digital health literacy is rooted in both "digital literacy" and "health literacy" [13, 98]. The digital health literacy skills framework also indicates that individual digital health behavior is primarily influenced by digital skills and health knowledge reserves [49, 76]. However, how this impact mechanism manifests in rural resident populations remains unclear. Therefore, the main objective of this paper is to elucidate the influence mechanism of digital literacy and health literacy on rural residents' participation in digital health behavior. By doing so, we aim to provide critical and in-depth insights into potential barriers to understanding digital health inequality between urban and rural areas. The research results can also offer important policy implications for narrowing the digital health gap between urban and rural areas, eliminating health inequality, and promoting better integration of rural residents into the digital health era.

## Research hypothesis

Digital health behaviors encompass the actions and habits of individuals utilizing digital platforms or devices, such as the Internet, Internet of Things, smartphones, wearable devices, and others, to enhance their health and improve their health status. These behaviors are multifaceted and can be broadly categorized into informational digital health behaviors (e.g., health information search, digital health management, etc.) and interactive digital health behaviors (e.g., online consultation, online medicine purchase, online health reviews, etc.). In the current digital landscape, participation in digital health behavior serves as a pivotal indicator of user integration into the digital health era. Previous literature indicates a correlation between users' participation in digital health behaviors and their digital health literacy, particularly emphasizing the foundational "digital literacy" and "health literacy" aspects [13, 38]. Accordingly, this paper constructs an empirical analysis framework incorporating users' digital health behavior participation, digital literacy, and health literacy. Through quantitative analysis of real data, this framework aims to clarify the impact mechanisms of digital literacy and health literacy on rural residents' participation in digital health behavior. This endeavor holds significant theoretical and practical implications for enhancing rural residents' access to digital health services, improving the overall health status of rural communities, and alleviating the health gap between urban and rural residents.

### Digital literacy's influence on digital health behavior among rural residents

In the realm of digital health, digital literacy, closely entwined with the concept, is a subject of considerable importance [52, 56]. Digital literacy encompasses the capacity of individuals to proficiently utilize digital tools for searching, acquiring, managing, integrating, evaluating, and analyzing digital resources within a specific life context, to construct new knowledge for constructive social action [4, 84]. In the era of accelerating social digitization, digital literacy is increasingly considered a prerequisite for meaningful participation in various facets of modern society, including life, study, and work [70, 74]. Previous studies indicate a keen interest among people in harnessing digital technologies to enhance healthcare delivery [18]. However, the effective promotion and widespread adoption of digital health applications in rural areas necessitate the possession of essential digital literacy by rural residents. Limited digital literacy weakens the confidence of rural residents in digital applications, potentially leading to digital health avoidance issues [97]. For many rural residents, digital technology remains abstract and complex. While numerous digital applications claim to have a "zero threshold," those based on intricate operations demand solid Internet operation skills, flexible digital thinking, and a profound understanding of the operational logic of the network society [8, 61, 65]. Failure to meet these requirements may result in negative digital health behaviors or even a complete avoidance of digital health participation. Li et al.'s [49] study found that individuals with higher digital literacy are more likely to actively search for digital health information and resources, consequently enhancing their health status based on the acquired health information. Moreover, it has been observed that improved health levels stimulate individuals' desire to further utilize digital health resources, prompting more positive digital health behaviors. Hence, an emerging body of literature underscores the imperative for governmental and decision-making entities to concentrate efforts on elevating the digital literacy of vulnerable populations. The aim is to bolster their confidence in utilizing digital health technologies, thereby fostering and advancing the adoption of such technologies [2, 3]. Building upon this, the paper posits the following hypothesis:

- *Hypothesis 1a: Digital literacy positively influences rural residents' participation in digital health behaviors.*

The concept of digital health behavior underscores the notion that user participation in this domain is multifaceted. Across various health scenarios, user participation in digital health behavior manifests diversely, encompassing, but not confined to, the informational and interactive digital health behaviors discussed earlier. In comparison to groups with low digital literacy, individuals possessing high digital literacy are both inclined and proficient in participating in a broad spectrum of digital health behaviors. For instance, within the realm of informational digital health behavior, such as health information searches, they actively compare diverse health information from multiple channels, including social networking platforms, instant messaging tools, and video streaming [20, 35]. Leveraging their information retrieval strategies, they adeptly navigate through redundant and complex health information to locate resources aligned with their needs [41, 69, 82]. Not confined to participation in informational digital health behavior, groups with high digital literacy also commonly exhibit active engagement in interactive digital health behavior, such as seeking health advice [41, 92]. Moreover, in scenarios like online medication purchases and online health reviews, individuals with high digital literacy demonstrate a robust willingness and ability to partake in digital health behavior. Building upon this, the paper posits the following hypothesis:

- *Hypothesis 1b: Digital literacy positively influences the diversity of rural residents' participation in digital health behaviors.*

### Health literacy's influence on digital health behavior among rural residents

Health literacy, defined as "the cognitive and social abilities determining an individual's access to, understanding of, and use of information to promote and maintain health" [51], assumes a critical role in rural contexts where medical resources are comparatively scarce. The internet, serving as a potent information retrieval tool, offers pivotal support to rural inhabitants in accessing abundant medical resources and quality health services [55]. However, low health literacy poses challenges in comprehending and adhering to guidelines and prescriptions conveyed by healthcare providers through digital platforms [61, 63]. This predicament negatively impacts the health status of rural residents, subsequently diminishing their inclination to participate in digital health behaviors [62]. Simultaneously, groups with restricted health literacy may disseminate misconstrued health information through online social platforms [10], creating conducive conditions for the proliferation of inaccurate health information. This poses a threat to the sustainability of the healthcare system, ultimately diminishing users' enthusiasm for participating in digital health behaviors. Fast et al. [26] highlighted that rural residents lacking health literacy encounter challenges in evaluating the reliability of digital health information. Enhancing rural residents' health literacy, as indicated by Okan et al. [60], fortifies their ability to comprehend and utilize digital health resources for health improvement, thereby elevating their likelihood of participating in digital health behaviors. Recent studies, exemplified by Zhou et al. [97], underscore the positive impact of improved health literacy in digital life on rural residents' utilization of medical services, a phenomenon termed the "health literacy effect." Building upon these insights, this paper posits the following hypothesis:

- *Hypothesis 2a: Health literacy positively influences rural residents' participation in digital health behaviors.*

Empirical investigations substantiate the intricate nexus between health literacy and pivotal dimensions of health dynamics, encompassing education, protection, prevention, and promotion [1, 11, 42]. Individuals endowed with elevated health literacy invariably manifest propitious health-related behaviors, exemplified in areas such as dietary habits, physical exercise, stress management, health responsibilities, and interpersonal relationships [41]. Recent scholarly inquiries have unveiled that within the digital health landscape, individuals possessing heightened health literacy are adept at navigating the online milieu to acquire health information. Additionally, they exhibit prowess in discerning, judging, and evaluating the accuracy, scientific validity, and efficacy of digital health information [49]. Hence, individuals endowed with elevated health literacy can adeptly and thoroughly employ health information to advance their personal health status. Furthermore, individuals with elevated health literacy display increased interest in health information derived from diverse sources such as social media, electronic magazines, and medical industry news websites [27]. They exhibit a willingness and proficiency in participating in various digital health behaviors, encompassing activities like online consultations, online medication purchases, health condition monitoring, and tracking, with the aim of enhancing their own or their family's health status [47]. Building upon these insights, this paper posits the following hypothesis:

- *Hypothesis 2b: Health literacy positively influences the diversity of rural residents' participation in digital health behaviors.*

### The influence of digital and health literacy interaction on rural residents' digital health behaviors

Drawing upon theoretical analysis and experiential synthesis concerning the interplay among digital literacy, health literacy, and digital health behavior among rural residents, it becomes evident that digital literacy predominantly encompasses proficiency in operating digital tools and skills, whereas health literacy is centered on the assessment, judgment, and application of health information. It is crucial to acknowledge that, for the majority of rural residents, navigating the Internet to seek health information tailored to their needs represents a complex and formidable undertaking [5, 68]. Simultaneously, the subsequent processes of comprehending, evaluating, and applying acquired health information demand a heightened level of health literacy. Previous literature reviews have underscored the pivotal role of digital literacy and health literacy as determinants influencing the extensive usage and acceptance of digital health applications [28, 50]. Consequently, the cultivation, enhancement, and fortification of digital literacy and health literacy among rural residents assume paramount importance. These endeavors hold the potential to support rural residents in effectively retrieving and applying pertinent health information and resources within the digital landscape. Furthermore, such initiatives contribute significantly to augmenting the likelihood of rural residents engaging in digital health behaviors [17].

Nevertheless, it is imperative to underscore that while digital literacy and health literacy fall within distinct categories of human capital capabilities, they intricately intersect and overlap functionally within the unified framework of users' participation in digital health behaviors [39]. Specifically, when an individual's health literacy is suboptimal, a robust digital literacy ensures widespread access to digital health services. Conversely, elevated levels of health literacy can supplant the need for extensive digital literacy attributes. For instance, a qualitative examination of arthritis patients revealed that individuals with sufficient health literacy demonstrated a propensity for seeking digital health information, even if their interest in "digital technology" was limited or their readiness for digital skills was modest [24].

Furthermore, the substitutive effects of digital literacy and health literacy become evident in their influence on the diversity of user participation in digital health behaviors. Rural residents with adequate digital literacy can adeptly navigate the intricate process of health information retrieval, even in the absence of prior health knowledge. It is crucial to underscore that the sense of accomplishment experienced by users, particularly vulnerable groups such as the elderly and those with lower incomes, upon overcoming internet challenges motivates them to explore a broader range of digital health solutions. This encompasses activities like online consultations, digital health management, online medication purchases, remote imaging diagnostics, and more. Indeed, digital literacy assumes a central role in the pursuit of diverse digital health solutions mentioned above and significantly substitutes for the functions of health literacy. Likewise, concerning the influence on the diversity of user participation in digital health behaviors, a high level of health literacy can also substitute for attributes or functions of digital literacy. Building upon these insights, this paper posits the following hypothesis:

- *Hypothesis 3a: Digital literacy and health literacy demonstrate an interaction effect on the participation of rural residents in digital health behaviors.*

- *Hypothesis 3b: Digital literacy and health literacy demonstrate an interaction effect on the diversity of participation in digital health behaviors of rural residents.*

## Data, variables, and models

### Source of data

#### Sample selection

The research team conducted a cross-sectional survey targeting rural residents in Zhejiang Province, China, spanning the period from August to November 2022. A multi-stage cluster random sampling method was employed to construct the study sample. In the initial stage, Zhejiang Province was segmented into five major blocks, namely North Zhejiang, East Zhejiang, West Zhejiang, South Zhejiang, and Central Zhejiang, according to the administrative divisions and local standards of Zhejiang Province. For the second stage, one county (or district) was randomly chosen from each of the five blocks, resulting in a total of five sample counties (or districts). Subsequently, in the third stage, one township (or town) was randomly selected from each of the sample counties (or districts), and a corresponding administrative village was chosen randomly within the selected township (or town). Finally, in the fourth stage, individuals aged between 18 and 85 years, residing in rural areas for more than 6 months annually, were randomly selected based on the roster provided by the administrative village. Exclusions from the study comprised rural residents with severe illnesses or those unable to respond to the survey questions.

#### Data collection tools and procedures

The study developed a structured questionnaire in Chinese to assess rural residents' participation in digital health behaviors, drawing extensively from relevant literature. The questionnaire encompassed key aspects, including the sociodemographic characteristics of rural residents, the manifestation of digital health behaviors, and measurement items for digital literacy and health literacy. Prior to actual data collection, a reliability pre-test was conducted. Five professionals specializing in medical informatics underwent a week-long training on the research objectives, content, subjects, and data collection procedures for this project. They subsequently executed tasks such as questionnaire distribution, on-site interviews, and data collection. Interviews took place in village meeting rooms or activity centers, organized under the guidance of professionals and supervised on-site. In the end, the project gathered pertinent data from 968 households of rural residents.

#### Sample characteristics

This study surveyed a total of five villages, each with an approximate sample size of 200 individuals. The gender distribution in the sample comprises approximately 21 males to 13 females. Participants in the study spanned ages from 18 to 85, with an average age of 54.814 years. The average educational attainment in the sample is 8.837 years, reflecting a junior high school educational level. The longest duration of education reported was 15 years, with 68 individuals having an education duration of three years or less, accounting for 7% of the sample. The average monthly family income is 4516 Chinese Yuan, and the average self-rated health status score is 2.997 on a five-point scale, indicating an above-average level. The proportion of married individuals in the sample is 89.36%, and the average family size is 3.2 people.

### Variables

#### Dependent variables

This paper, combining the "Context" conceptual framework [71], builds on the concept of digital health behavior and references the Internet health behavior classification system proposed by Hale et al. [30]. Starting from the behavioral context, the most typical digital health behaviors of rural residents are categorized into the following four aspects:

1. Health Information Search Behavior: This category primarily encompasses the actions undertaken by rural residents in searching, retrieving, and utilizing pertinent health or medical information in the digital realm. Examples include searching for drug side effects, exploring nutritional information about healthy health foods, and understanding a spectrum of health indicators.
2. Digital Health Management Behavior: This facet pertains to the endeavors of rural residents to manage their health by leveraging digital platforms or devices. Activities include online inquiries into physical examination results, querying medical reports, and engaging in health management practices facilitated by digital wearable devices.
3. Online Health Consultation Behavior: This domain involves the actions of rural residents on online health portals and mobile medical platforms. Examples encompass seeking medical advice, engaging in self-diagnosis and consultation, and participating in health consultations through platforms such as Dingxiang Doctor, Ping An Good Doctor, and Chunyu Doctor, among others.
4. Internet-based medication purchase behavior: This dimension encapsulates the conduct of rural residents acquiring drugs (medicines, health foods, medical supplies, etc.) through online platforms such as Ali Health, JD Health, and Meituan Buy Medicine.

In accordance with the previously posited research hypotheses, this paper considers "Digital Health Behavior Participation of Rural Residents"(DH_behavior) and "Diversity of Digital Health Behavior Participation of Rural Residents"(DH_behavior_ diversity) as dependent variables. The notation "1" or "0" is employed to represent "Digital Health Behavior Participation of Rural Residents." Specifically, within the digital health experiences of rural residents, a value of "1" is assigned if they have participated in at least one of the following behaviors: "health information search behavior," "digital health management behavior," "online health consultation behavior," or "internet-based medication purchase behavior"; otherwise, a value of "0" is assigned. The variable "Diversity of Digital Health Behavior Participation of Rural Residents" assumes values of 0, 1, 2, 3, or 4. Consequently, if rural residents have partaken in all the specified digital health behaviors, the dependent variable is assigned a value of 4. If they have engaged in three of these behaviors, the dependent variable's value is 3, and so forth, following a similar pattern for other scenarios.

### Key explanatory variables

The key explanatory variables in this study encompass the digital literacy and health literacy of rural residents. To assess the digital literacy of rural residents, this study utilized the Digital Literacy Measurement Scale for Rural Residents developed by Chinese scholars [78]. Additionally, it referred to Qian [65] and the European Union Digital Competence Framework DigComp 2.1 since 2013 for pertinent items related to user digital literacy. The study crafted an evaluation index system for the digital literacy level of rural residents, encompassing 13 items distributed across five dimensions: "digital general literacy," "digital social literacy," "digital search literacy," "digital creative literacy," and "digital security literacy," as delineated in Appendix 1. Subsequently, based on real data, a factor analysis was conducted on the scale. The results of the factor analysis revealed a Kaiser–Meyer–Olkin (KMO) value of 0.885 in the adequacy test, underscoring a robust correlation between the measurement items of rural residents' digital literacy. Concurrently, the significance *p*-value of the Bartlett sphericity test statistic was 0.000, affirming the efficacy of the factor analysis results. Following the computation, the Cronbach's alpha coefficient for all measurement items in this scale was 0.874, and the Cronbach's alpha coefficients for each sub-dimension were all higher than 0.85, indicative of the commendable measurement reliability of the items. Moreover, the factor loading values for each measurement item in this scale were all greater than 0.5, attesting to the robust convergent validity of the variable measurements.

When assessing the health literacy of rural residents, this study drew upon prior research on user health literacy measurement [14, 52, 75] and the survey questionnaire "66 Items of Chinese Residents' Health Literacy," endorsed by the National Health Commission in 2008. To streamline the measurement process for simplicity and efficiency, this study selected 10 items from three dimensions: "health philosophy," "health knowledge," and "health skills" to craft an evaluation index system for the health literacy level of rural residents, as elaborated in Appendix 2. In the factor analysis results, the Kaiser–Meyer–Olkin (KMO) value for sample adequacy testing was 0.761, indicating a commendable correlation between the measurement items of rural residents' health literacy. Concurrently, the significance *p*-value of the Bartlett sphericity test statistic was 0.000, affirming the efficacy of the factor analysis results. Although the scale is not strictly a Likert scale, to validate the reliability of the modified scale, the study randomly divided the sample questionnaire data into two equal parts (*n* = 968/2 = 484) for exploratory factor analysis and confirmatory factor analysis. Utilizing principal component analysis and maximum variance rotation for exploratory factor analysis, a total of 3 factors with eigenvalues surpassing 1 were extracted, elucidating a cumulative variance of 71.730%, with contribution rates of 42.615%, 18.257%, and 10.858%, respectively. Furthermore, the Cronbach's alpha coefficients for each item exceeded 0.84, and the factor loadings of all measurement items exceeded 0.4. Another set of data (*n* = 484) underwent confirmatory factor analysis. The results revealed that the chi-square value of the scale was 642.515, with a degree of freedom of 168, and their ratio was less than 5. Meanwhile, the values of RMSEA, SRMR, and CFI all indicate that the scale is acceptable.

This study utilizes both factor analysis and the summation method to evaluate the digital literacy and health literacy of rural residents. In the factor analysis approach, the weight of each factor's score is determined by its proportional contribution to the cumulative variance contribution rate, which is subsequently employed to calculate the overall level. The summation method involves algebraically summing the scores provided by rural residents for each question to derive a comprehensive total score. The results obtained through the summation method are then subjected to robustness testing.

### Control variables

This study systematically incorporates control variables derived from the multifaceted dimensions of individual, family, and geographical characteristics of rural residents [36, 44, 49, 98]. In addition, regional fixed effects at the village level are meticulously controlled. The precise definitions, assignments, and descriptive statistical outcomes of these variables are delineated in Table 1.

**Table 1:** Definition of variables, variable assignment, and descriptive statistics results

Items	Variables	Variable Value	Mean	Standard Deviation	Minimum Value	Maximum value	Nature of Variables
Dependent Variable	Health Information Search Behavior	If participating, its value is 1; otherwise, its value is 0	0.435	0.496	0	1	Categorical Variables
	Digital Health Management Behavior	If participating, its value is 1; otherwise, its value is 0	0.228	0.420	0	1	Categorical Variables
	Online Health Consultation Behavior	If participating, its value is 1; otherwise, its value is 0	0.151	0.358	0	1	Categorical Variables
	Internet-based medication purchase behavior	If participating, its value is 1; otherwise, its value is 0	0.118	0.323	0	1	Categorical Variables
	DH_behavior	A value of 1 is assigned if any of the aforementioned behaviors occur; otherwise, the value is 0	0.468	0.499	0	1	Categorical Variables
	DH_behavior_diversity	Summing the values of the aforementioned four behaviors	0.930	1.239	0	4	Categorical Variables
Key Explanatory Variables	Digital Literacy 1	Calculated digital literacy based on factor analysis and applied the 3-sigma rule to eliminate negative values	1.100	0.787	0.002	2.590	Continuous Variables
	Digital Literacy 2	The direct summation of scores for each item in digital literacy	7.254	3.485	0	13	Continuous Variables
	Health Literacy 1	Calculated health literacy based on factor analysis and applied the 3-sigma rule to eliminate negative values	16.500	6.297	0.015	33.646	Continuous Variables
	Health Literacy 2	The direct summation of scores for each item in health literacy	10.217	3.312	2	19	Continuous Variables
Main Control Variables	Gender	Female = 0, Male = 1	0.624	0.485	0	1	Categorical Variables
	Age	Actual survey value (unit: years)	54.814	12.925	18	85	Continuous Variables
	Marital Status	Married = 1, Unmarried = 0	0.894	0.309	0	1	Categorical Variables
	Years of Education	Actual survey value (Unit: years)	8.837	2.699	0	15	Continuous Variables
	Self-rated Health	Extremely poor = 1; Poor = 2; Average = 3; Good = 4; Excellent = 5	2.997	0.960	1	5	Categorical Variables
	Family Size	Actual survey value (unit: individuals)	3.276	1.007	2	6	Categorical Variables
	Average Monthly Family Income	Actual survey value (unit: yuan)	4516.967	2329.139	0	12,000	Continuous Variables
	Distance to the Nearest Healthcare Facility	Actual survey value (unit: kilometers)	2.502	0.475	1.5	3.8	Continuous Variables
Village Characteristics	A Village, B Village, C Village, D Village, E Village	A = 1; B = 2; C = 3; D = 4; E = 5	3.200	1.300	1	5	Categorical Variables
Instrumental Variables	Instrumental Variable 1	Values computed based on the specified algorithm	1.094	0.374	0.729	1.759	Continuous Variables
	Instrumental Variable 2		10.168	3.131	4.990	14.946	Continuous Variables

### Econometric model

To test the hypothesis mentioned earlier, this study, considering the types and characteristics of the dependent variables, namely, the binary nature of "digital health behavior participation of rural residents" (0 or 1) and the count nature of "diversity of digital health behavior participation of rural residents" (0, 1, 2, 3, 4), and drawing from Zheng et al.'s work [96], constructs targeted Probit and Poisson models. These models are designed to examine the influence of rural residents' digital literacy, health literacy, and the interaction between the two on rural residents' participation in digital health behavior and the diversity of digital health behavior participation. The general model is represented as follows:

1 $$prob(DH\_behavior_{i} ) = \alpha_{0} + \beta_{1} dig\_liter_{i} + \beta_{2} hea\_liter_{i} + \beta_{3} X_{i} + \mu_{i} + \varepsilon_{i}$$

2 $$pois(DH\_behavior\_diversity_{i} = n_{i} \left| {dig\_liter_{i} ,hea\_liter_{i} ,X_{i} } \right.) = {{e^{{ - \lambda_{i} }} \lambda_{i}^{{n_{i} }} } \mathord{\left/ {\vphantom {{e^{{ - \lambda_{i} }} \lambda_{i}^{{n_{i} }} } {n_{i} }}} \right. \kern-0pt} {n_{i} }}!$$

In this context, $DH\_behavior_{i}$ serves as the dependent variable in the study. A $DH\_behavior_{i}$ value of 1 signifies rural residents' participation in digital health behaviors, while a $DH\_behavior_{i}$ value of 0 indicates non-participation in such behaviors. $DH\_behabvior\_diversity_{i}$ denotes the diversity of rural residents' involvement in digital health behaviors, where n takes values 0, 1, 2, 3, 4. $\lambda$ is greater than 0 and is a constant. $dig\_liter_{i}$ and $hea\_liter_{i}$, the central explanatory variables in this research, symbolize the digital literacy and health literacy of rural resident $i$, respectively. $X_{i}$ constitutes the control variable, encompassing various factors influencing the digital health behaviors of rural residents, specifically encompassing individual characteristics such as gender, age, marital status, years of education, and health status; family characteristics including family size and average monthly income; geographical characteristics like distance to the nearest medical institution. Regional fixed effects are represented by $\mu_{i}$, while $\varepsilon_{i}$ denotes the random disturbance term accounting for unobservable factors, adhering to a standard normal distribution. Parameters to be estimated are denoted as $\beta_{1\sim 3}$.

Building upon the aforementioned considerations, this study extends its investigation by incorporating the interaction term of rural residents' digital literacy and health literacy into both the Probit and Poisson models. This extension aims to explore the nuanced impact of the interaction between digital literacy and health literacy on the digital health behaviors of rural residents. The general formulation of the model is articulated as follows:

3 $$prob(DH\_behavior_{i} ) = \alpha_{0} + \beta_{1} dig\_liter_{i} + \beta_{2} hea\_liter_{i} + \beta_{3} dig\_liter_{i} \times hea\_liter_{i} { + }\beta_{4} X_{i} + \mu_{i} + \varepsilon_{i}$$

4 $$pois(DH\_behavior\_diversity_{i} = n_{i} \left| {dig\_liter_{i} ,hea\_liter_{i} ,dig\_liter_{i} \times hea\_liter_{i} ,X_{i} } \right.) = {{e^{{ - \lambda_{i} }} \lambda_{i}^{{n_{i} }} } \mathord{\left/ {\vphantom {{e^{{ - \lambda_{i} }} \lambda_{i}^{{n_{i} }} } {n_{i} }}} \right. \kern-0pt} {n_{i} }}!$$

Within this context, $dig\_liter_{i} \times hea\_liter_{i}$ represents the interaction term involving the digital literacy and health literacy of rural residents. This term is pivotal for elucidating the intricate relationship between digital literacy, health literacy, and the digital health behaviors of rural residents under the influence of their interaction. The parameter $\beta_{4}$ is the estimated coefficient associated with the interaction term. A negative $\beta_{4}$ suggests a interaction effect between digital literacy and health literacy in shaping the digital health behavior of rural residents, while a positive $\beta_{4}$ implies a complementary effect.

## Regression results and analysis

Table 2 presents the influence of digital literacy, health literacy, and their interaction on the digital health behavior of rural residents. As depicted in columns (1) and (2) of Table 2, the estimated coefficients for digital literacy are 0.502 and 0.245, respectively, both demonstrating statistical significance at the 1% level. This underscores the substantial impact of digital literacy on rural residents' participation in digital health behavior. Consequently, digital literacy not only heightens the likelihood of rural residents participating in digital health behavior but also broadens the diversity of their involvement, encompassing more scenarios and exhibiting a more extensive range of digital health behaviors. Thus, affirming hypotheses 1a and 1b.

**Table 2:** Baseline regression results of digital literacy, health literacy, and rural residents' participation and diversity of participation in digital health behaviors

Variable	(1)	(2)	(3)	(4)
	**Participation**	**Diversity**	**Participation**	**Diversity**
	***Probit model***	***Poisson model***	***Probit model***	***Poisson model***
Digital Literacy	0.502***	0.245***	0.536***	0.289***
	(0.060)	(0.029)	(0.060)	(0.027)
Health Literacy	0.015	0.018***	0.027**	0.050***
	(0.013)	(0.005)	(0.011)	(0.006)
Digital Literacy # Health Literacy			-0.045***	-0.040***
			(0.017)	(0.004)
Gender	0.257***	0.129***	0.243***	0.109***
	(0.050)	(0.028)	(0.048)	(0.023)
Age	0.125	0.130*	0.146	0.100**
	(0.145)	(0.070)	(0.137)	(0.050)
Marital Status	0.125*	0.081*	0.113*	0.052
	(0.068)	(0.041)	(0.067)	(0.032)
Years of Education	0.486***	0.181***	0.501***	0.171***
	(0.128)	(0.058)	(0.127)	(0.046)
Self-rated Health	0.053*	0.013	0.056*	0.013
	(0.031)	(0.016)	(0.030)	(0.012)
Family Size	0.003	0.005	0.002	0.002
	(0.028)	(0.012)	(0.027)	(0.008)
Average Monthly Family Income	0.549***	0.546***	0.486***	0.378***
	(0.068)	(0.046)	(0.067)	(0.045)
Distance to the Nearest Healthcare Facility	0.025	-0.065**	0.034	-0.048**
	(0.066)	(0.030)	(0.067)	(0.023)
Regional Dummy Variable	Control	Control	Control	Control
Wald	255.97***	922.08***	280.87***	1187.01***
Pseudo R2	0.6645	0.3931	0.6728	0.4124
Sample Size	968	968	968	968

^*^, **, *** denote significance at the 10%, 5%, and 1% levels, respectively; reported in the table are marginal effects, with robust standard errors in parentheses

On a different note, concerning the influence on rural residents' participation in digital health behavior, the estimated coefficient for health literacy is 0.015, lacking statistical significance. However, regarding the impact on the diversity of rural residents' participation in digital health behavior, the estimated coefficient for health literacy is 0.018, signifying significance at the 1% level. This suggests that heightened health literacy among rural residents corresponds to more extensive participation in digital health behavior. Consequently, hypothesis 2b finds empirical support.

Based on the findings presented in columns (1) and (2) of Table 2, several conclusions emerge. Firstly, it is not an overstatement to assert that digital literacy is evolving into a crucial survival skill in the era of the digital economy [67, 72]. In the rapidly transforming digital society, users' participation in digital health behavior transcends the passive receipt of health information [45]. Instead, users grapple with diverse and intricate digital health scenarios, encompassing activities such as searching for health information, managing health digitally, seeking online health consultations, and making online drug purchases [29]. These scenarios demand not only a foundational level of digital literacy for basic information interaction but also a sophisticated level of digital literacy for diagnostic or treatment-related activities through online platforms [86]. Consequently, when rural residents necessitate treatment due to illness or participate in health consultations, health management, and other digital health activities for health-related purposes, digital literacy significantly and positively influences their participation in digital health behavior.

Secondly, the influence of health literacy on the digital health behavior of rural residents manifests more prominently in terms of its diversity. The essence of health literacy lies in individuals' ability to acquire and comprehend health information, subsequently applying the acquired knowledge to make decisions and adopt behaviors conducive to enhancing their health status [35]. Nonetheless, elevated health literacy does not necessarily correlate with residents' participation in digital health behaviors, rather, it is more likely to be associated with commonplace offline medical scenarios, such as purchasing medication from pharmacies and seeking medical attention at hospitals [21, 83]. Hence, this likely explains why health literacy does not emerge as a significant predictor of rural residents' participation in digital health behavior. Notably, health literacy does exhibit a substantial positive influence on the diversity of rural residents' participation in digital health behavior. This can be attributed to individuals with higher health literacy levels possessing more efficient skills in obtaining and comprehending online health information, coupled with their ability to discern the correctness, scientific validity, and effectiveness of such information [49, 79]. Individuals exhibiting elevated levels of health literacy demonstrate a proclivity for directing their attention toward diverse outlets of health-related information. This inclination serves to augment both the probability and impetus for these individuals to delve into and embrace various measures directed at enhancing personal well-being [21, 79]. Importantly, these health promotion endeavors transcend the conventional realm of offline medical services and encompass the domain of more streamlined and efficient digital health service scenarios. Consequently, this heightened health literacy may propel rural residents towards active participation in a spectrum of digital health behaviors, including but not limited to health information retrieval and digital health management.

As evidenced by the findings in columns (3) and (4) of Table 2, the estimated coefficients for the interaction terms of digital literacy and health literacy are -0.045 and -0.040, both significant at the 1% level. This indicates a diminishing positive impact of digital literacy on rural residents' participation in digital health behaviors as their health literacy improves. Similarly, with the enhancement of rural residents' digital literacy, the positive promoting effect of health literacy on the diversity of their participation in digital health behaviors weakens. Consequently, concerning the impact on rural residents' participation and its diversity, a significant substitution relationship is observed between digital literacy and health literacy, confirming Hypothesis 3.

Despite digital literacy and health literacy falling into distinct categories of human capital, their functional overlap in influencing rural residents' digital health behavior offers a plausible explanation for the interaction effect. We can infer that in the digital realm, individuals with adequate digital literacy are inclined to focus on diverse digital health information sources, employing advanced digital skills for tasks such as information retrieval, processing, online medical consultations, and even engaging in health services utilizing digital therapy, even in the absence of specific health knowledge [7, 59]. Similarly, individuals with proficient health literacy exhibit competence in accessing, comprehending, evaluating, and applying health information in a digital environment, utilizing digital capabilities for tasks related to healthcare, disease prevention, and health promotion [75]. Throughout this process, the positive driving effect of digital literacy on individuals' participation in digital health behaviors is supplanted by adequate.

Furthermore, the coefficients of certain control variables in Table 2 hold noteworthy economic implications. The regression results reveal a significantly positive marginal coefficient of gender on the impact of rural residents' participation in digital health behaviors and the diversity of their participation, signifying that, in comparison to women, men are more inclined to participate in digital health behaviors. This propensity may stem from men's greater openness to embracing innovations and seeking convenience, rendering them more prone to embracing and utilizing digital health services [89]. Similarly, the significantly positive marginal coefficients of years of education and average monthly family income, at the 1% significance level, indicate that rural residents with extended educational backgrounds and higher average monthly family incomes are more predisposed to adopting digital health services and subsequently engaging in digital health behaviors [25]. This aligns with the observed reality wherein individuals with lower education levels and incomes in rural areas often face pronounced "digital divide" and "knowledge gap" phenomena, coupled with limitations imposed by the "information cocoon" effect, making them less likely to partake in digital health behaviors compared to their counterparts with higher education levels and household incomes [12, 43]. This is consistent with the knowledge gap hypothesis [81]. Intriguingly, there was no discernible correlation between the age of rural residents and their participation in digital health behaviors. This may be attributed to divergent physical conditions and attitudes towards health services between younger and older demographic groups.

## Endogeneity discussion

Given that the pivotal explanatory variables in this study originate from factor analysis, inherent computational inaccuracies are inescapable. Concurrently, it is essential to recognize the potential for reverse causality between digital literacy, health literacy, and the participation of rural residents in digital health behaviors. Moreover, the omission of certain explanatory variables in the model introduces a potential source of endogeneity, raising concerns about the reliability of the regression results. To address these challenges, this study employs the instrumental variable method to mitigate endogeneity issues, contingent upon the correlation condition and exogenous requirement.

The instrumental variables for digital literacy and health literacy in the surveyed samples, designated as Instrumental Variable 1 and Instrumental Variable 2 respectively, were identified as follows: the "average digital literacy value among other samples residing in the same village, excluding the respondents themselves," and the "average health literacy value among other samples residing in the same village, excluding the respondents themselves." Subsequently, the instrumental variable method was employed to estimate the specified model. The rationale for selecting these instrumental variables is grounded in the similarity of the digital environment within the same village, indicating that an individual's digital literacy is influenced by the average level of others in the same village. Additionally, the digital health behavior engagement of the surveyed individuals is not directly correlated with the digital literacy of others. Theoretical adherence to the requirements of correlation and exogeneity justifies the choice of these instrumental variables. Similar reasoning guided the selection of instrumental variables for health literacy.

The regression outcomes of both the IVProbit and IVPoisson models are presented in Table 3. Firstly, without assuming a distribution and when constraints are nonlinear, it is imperative to establish the overall significance of the entire model through the Wald test. The results reveal that the Wald test values for Models (1) to (4) are all significantly non-zero at the 1% statistical level. This signifies a robust overall fit of the models, warranting further analysis [95]. Secondly, the exogeneity of the explanatory variables is assessed through the significance of the Wald test of exogeneity. The findings indicate that the Wald test of exogeneity is significantly non-zero at the 1% statistical level, rejecting the null hypothesis of "all explanatory variables being exogenous." This suggests the effectiveness of employing the instrumental variable method to address potential endogeneity issues in the model. Thirdly, with the introduction of instrumental variables, evaluating whether these instruments are "weak instruments" becomes crucial. Utilizing the Cragg-Donald Wald F-statistic proposed by Cragg and Donald [16] and the critical values provided by Stock and Yogo [77], the results demonstrate that the Cragg-Donald Wald F-statistic is 441.02, surpassing the critical value of 4.58 for 15% and also exceeding the critical value of 7.03 for 10%. This implies that the selected instrumental variables in this study are not deemed "weak instruments." Additionally, the "weak instruments" test, conducted using the external command "weakiv" in Stata, reinforces that the above instruments are not considered "weak instruments." Finally, the consistency observed across the results of the IVProbit model, IVPoisson model, and the baseline regression underscores the robustness of the findings.

**Table 3:** Instrumental variable estimation results

Variable	IVProbit			IVPoisson
	**First-Stage regressions**		**Instrumental variables regressions**	**/**
	**Digital Literacy**	**Health Literacy**	**Participation**	**Diversity**
	**(1)**	**(2)**	**(3)**	**(4)**
Instrumental Variable 1	0.988***(0.033)			0.988***(0.034)
Instrumental Variable 2		0.951***(0.016)		0.951***(0.020)
Digital Literacy			1.984***(0.183)	2.298***(0.330)
Health Literacy			0.004(0.037)	0.061***(0.023)
Control Variables	Control	Control	Control	Control
Regional Dummy Variable	Control	Control	Control	Control
Exogeneity Wald Test	43.23***			/
Wald	394.02***		43.23***	1398.55***
First-Stage F-value	436.478***	1196.4***		
Weak Instrument Variable Test	Cragg-Donald Wald F = 441.02 Stock-Yogo weak ID test critical values(10%) = 7.03			
	AR = 130.14*** & Wald = 117.85*** (Derived using the “weakiv” command)			
Sample Size	968	968	968	968

*, **, *** denote significance at the 10%, 5%, and 1% levels, respectively; values in parentheses represent robust standard errors

## Robustness testing

To ascertain the robustness of the earlier estimation results, this study employs the "summation method" to compute the outcomes of digital literacy and health literacy, substituting the previously utilized factor score method for the re-regression. The results are delineated in Table 4. The outcomes of model (1) and model (2) reveal that the estimated coefficients of digital literacy are significantly positive at the 1% level. Moreover, the estimated coefficient depicting the influence of health literacy on the diversity of rural residents' participation in digital health behavior is significantly positive at the 1% level. Model (3) and model (4) present regression results that incorporate the interaction terms of digital literacy and health literacy, with their estimated coefficients being significantly negative at the 1% level. It is noteworthy that despite the change in measurement methods for digital literacy and health literacy, the regression results consistently align with the earlier conclusions, underscoring the robustness of the primary findings in this study.

**Table 4:** Robustness test: digital literacy, health literacy, and rural residents' participation and diversity of participation in digital health behaviors

Variable	(1)	(2)	(3)	(4)
	**Participation**	**Diversity**	**Participation**	**Diversity**
	***Probit model***	***Poisson model***	***Probit model***	***Poisson model***
Digital Literacy	0.131***	0.078***	0.135***	0.081***
	(0.014)	(0.008)	(0.013)	(0.006)
Health Literacy	0.017	0.018***	0.031***	0.050***
	(0.013)	(0.005)	(0.011)	(0.005)
Digital Literacy # Health Literacy			-0.011***	-0.011***
			(0.004)	(0.001)
Control Variables	Control	Control	Control	Control
Regional Dummy Variable	Control	Control	Control	Control
Wald	255.46***	975.65***	270.57***	1121.89***
Pseudo R2	0.6711	0.4023	0.6781	0.4213
Sample Size	968	968	968	968

*, **, *** denote significance at the 10%, 5%, and 1% levels, respectively; reported in the table are marginal effects, with robust standard errors in parentheses

## Heterogeneity analysis

The regression results detailing the influence of digital literacy, health literacy, and their interaction on various digital health behaviors among rural residents are presented in Table 5. Notably, the estimated coefficient for digital literacy is consistently and significantly positive at the 1% level for all digital health behaviors, reaffirming hypothesis 1a. However, the impact of health literacy on distinct digital health behaviors of rural residents exhibits variation. Specifically, the influence of health literacy on health information search behavior is not statistically significant. Nonetheless, for the remaining three digital health behaviors, the estimated coefficient of health literacy is significantly positive at the 1% significance level, thus confirming hypothesis 2a in specific scenarios. Additionally, through a comparison of the magnitudes of the marginal coefficients, it can be inferred that digital literacy plays a more pivotal role in predicting the participation and diversity of digital health behaviors among rural residents.

**Table 5:** The influence of digital literacy, health literacy, and their interaction on various digital health behavior participation among rural residents

Variable	(1)	(2)	(3)	(4)	(5)	(6)	(7)	(8)
	**Participation**							
	**Health Information Search Behavior**	**Digital Health Management Behavior**	**Online Health Consultation Behavior**	**Internet-based medication purchase behavior**	**Health Information Search Behavior**	**Digital Health Management Behavior**	**Online Health Consultation Behavior**	**Internet-based medication purchase behavior**
Digital Literacy	0.392*** (0.050)	0.059*** (0.017)	0.039*** (0.013)	0.022*** (0.010)	0.419*** (0.054)	0.060*** (0.016)	0.038*** (0.013)	0.125*** (0.021)
Health Literacy	-0.003 (0.006)	0.010*** (0.002)	0.003*** (0.001)	0.003*** (0.001)	0.006 (0.005)	0.010*** (0.002)	0.003*** (0.001)	0.016*** (0.002)
Digital Literacy # Health Literacy					-0.019** (0.007)	-0.003* (0.002)	-0.0004 (0.001)	-0.007*** (0.002)
Control Variables	Control	Control	Control	Control	Control	Control	Control	Control
Regional Dummy Variable	Control	Control	Control	Control	Control	Control	Control	Control
Wald	262.68***	189.97***	187.24***	149.06***	290.71***	214.85***	205.30***	131.65***
Pseudo R2	0.5731	0.4809	0.4338	0.4852	0.5827	0.4848	0.4341	0.4984
Sample Size	968	968	968	968	968	968	968	968

*, **, *** denote significance at the 10%, 5%, and 1% levels, respectively; reported in the table are marginal effects, with robust standard errors in parentheses

The outcomes from Model (5) to Model (8) reveal variations in the significance of the estimation coefficients for interaction terms across distinct digital health behaviors. For health information searching, digital health management, and internet drug purchasing behaviors, there is a clear substitutive relationship between the digital literacy and health literacy of rural residents. However, in the case of online health consultation behavior, although the interaction effect of digital literacy and health literacy is not statistically significant, the negative interaction coefficient suggests an existing substitution relationship between digital literacy and health literacy in influencing the digital health behavior of rural residents. This further validates Hypothesis 3a and 3b.

## Discussion and conclusions

With the confluence of digital technology and healthcare, there is a growing body of research exploring the interplay between digital literacy, health literacy, and residents' participation in digital health behavior [39, 49, 88]. However, existing literature exhibits certain limitations: Initially, the extant literature primarily provided conceptual elucidations of the functions and impacts of digital literacy and health literacy [22, 57]. However, it fell short in constructing an empirical analytical framework that integrates digital literacy, health literacy, and digital health behaviors within diverse digital health scenarios. This deficiency has contributed to a dearth of scientifically rigorous findings. Secondly, amid the deepening digital transformation of the economy and society, remote rural areas facing geographical disadvantages risk being neglected and marginalized [66, 85]. The resultant health inequalities between urban and rural areas demand urgent attention and resolution. However, current studies often overlook the digital health behaviors of rural residents. Against this backdrop and in alignment with the ongoing digital transformation of rural public health systems in the era of the digital economy, this paper establishes an evaluation index system for digital literacy and health literacy among rural residents. Drawing on survey data from 968 respondents in five administrative villages in Zhejiang Province, the study provides a micro-level analysis of the digital health behaviors of rural residents. Employing Probit and Poisson models, the paper empirically investigates the effects of digital literacy and health literacy, along with their interaction terms, on rural residents' responses to digital health behavior and the diversity of their participation. This quantitative approach unveils the dynamic role of digital literacy and health literacy. Furthermore, instrumental variable methods, robustness tests, and heterogeneity analyses are employed to bolster the robustness of the conclusions.

### Principal findings

This study delves into various digital health behaviors, encompassing health information searching, digital health management, online health consultation, and Internet drug purchasing. The findings affirm that digital literacy serves as a pivotal predictor of rural residents' participation in digital health behaviors, a conclusion supported by extensive research [3, 32, 94]. Additionally, this investigation unveils, for the first time, that digital literacy contributes to broadening the diversity of rural residents' involvement in digital health behaviors. In other words, individuals with heightened digital literacy are more inclined to explore a diverse array of digital health measures to enhance their well-being. While prior literature has not explicitly explored the " diversity of digital health behavior" perspective, analogous conclusions have been suggested by scholars who posit that user digital literacy significantly influences the extensive utilization and acceptance of health information systems [22]. These health information systems encompass various digital health behaviors, such as health information searches and health status management and tracking.

In contrast to digital literacy, health literacy emerges as a non-significant predictor of rural residents' participation in digital health behaviors. This observation may stem from the fact that heightened health literacy propels individuals to utilize their accrued health knowledge for decision-making or behaviors geared toward improving their health status [35]. However, these behaviors may not necessarily manifest as digital health activities, rather, they might be more prevalent in conventional offline health scenarios, such as purchasing medicine from a pharmacy or seeking medical assistance in a hospital. This discovery aligns with the outcomes of Manganello et al. In their prior cross-sectional telephone survey involving 1,350 individuals in New York State, self-reported health literacy did not emerge as a robust predictor of digital health behaviors [51]. It is crucial to acknowledge, though, that a study focused on primary care patients discovered a significant association between health literacy and participation in digital health behaviors [9], contradicting our results. The disparities could be attributed to variations in study populations or the use of different health literacy measurement tools, emphasizing the need for a comprehensive exploration of the relationship between health literacy and digital health behaviors across diverse cohorts.

Significantly, this study unveils, for the first time, that health literacy plays a pivotal role in predicting the diversity of participation in digital health behaviors among rural residents. This phenomenon may be attributed to individuals with elevated health literacy exhibiting a heightened capacity to attentively navigate health information from diverse sources. Their effectiveness in discerning the accuracy, scientific validity, and credibility of online health information may further motivate them to actively pursue various measures and choose from a wider array of digital scenarios to enhance their health. Supporting this notion, a recent cross-sectional study examining the correlation between health literacy and digital health behavior among older Chinese adults indirectly corroborates our findings [49].

This study uncovers a novel insight into the substantial interaction effect resulting from the interaction term of digital literacy and health literacy on rural residents' participation and diversity of participation in digital health behaviors. Conceptually, digital literacy is defined as "the ability of individuals to use digital tools to search for, acquire, manage, integrate, evaluate, and analyze digital resources in a given context to build new knowledge for constructive social action" [4, 84]. On the other hand, health literacy encompasses "the cognitive and social abilities that determine an individual's access to, understanding of, and use of information to promote and maintain health" [51]. In the realm of health management, digital literacy and health literacy converge in their shared goal of "obtaining, understanding, and using information to achieve the goal of improving one's health." Consequently, a proficient level of either digital literacy or health literacy is often closely linked to positive health outcomes across various health domains [23]. In light of this, reinforcing the digital literacy and health literacy of rural residents holds significant promise in enhancing their health status and mitigating health disparities between urban and rural areas, particularly in the context of the digital age.

This study further unveiled a moderating impact of socioeconomic factors on the engagement in digital health behaviors and the diversity thereof among rural residents. Specifically, individuals in rural areas with extended educational durations and elevated average monthly incomes exhibit a higher propensity to embrace digital health services. This concurs with the research findings of scholars like Van et al. [87] and Vainieri et al. [85]. To a certain extent, this conclusion underscores the "resource reinforcement" effect, suggesting that individuals with heightened social status are more inclined to access diverse resources, including digital skills training, health knowledge, and health education resources.

### Further discussion

In the digital health era, electronic health services utilizing the internet, smartphones, wearable devices, and health portals are pivotal solutions ensuring health security across age groups and geographical regions. Consequently, individuals need to possess fundamental digital literacy and health literacy to meaningfully engage and attain optimal health and well-being in an increasingly digital society propelled by information and communication technology. This need is particularly pronounced for rural residents in regions with limited social resources and remote settings [40, 88]. Rural residents equipped with sufficient digital literacy are more inclined to leverage digital tools for health enhancement, demonstrating heightened awareness of digital health technologies. Those with advanced digital literacy can proficiently operate health devices using digital interfaces, access electronic health records through mobile applications, manage personal health, participate in real-time health exchanges with healthcare providers or peers through social media networks, and seamlessly communicate with remote healthcare providers. Meanwhile, the significance of health literacy is self-evident, as extensive prior research has consistently shown its close association with health-related factors such as health behavior, disease management, and quality of life [1, 11, 41]. In the digital health landscape, rural residents possessing adequate health literacy demonstrate the ability to engage in diverse digital health behaviors, exhibiting interest in accessing a broader array of health information sources. They are equipped to search for, comprehend, evaluate, and apply acquired health knowledge to make informed health-related decisions [11, 97]. This proficiency enables them to judiciously allocate high-quality health resources, thereby sustaining and enhancing their health [80].

### Suggestions

Regrettably, a substantial digital health divide persists between rural and urban residents. In response, this study advocates for governments in China and globally to prioritize initiatives aimed at "enhancing the digital literacy of rural residents, reinforcing health education, and fostering ongoing development in health literacy." This imperative should assume a central role in the public health sector, encompassing strategic actions such as the formulation of policy documents promoting "digital adaptation in rural areas," augmentation of integrated digital infrastructure in both urban and rural domains, and allocation of dedicated resources for customized digital skills training programs targeting rural residents. Concurrently, to elevate the health literacy of rural residents, endeavors should be undertaken to integrate health education into rural and community settings. This may involve the distribution of "health literacy pamphlets" and the establishment of health knowledge dissemination columns. The implementation of these initiatives holds the potential to ameliorate the digital health gap between urban and rural areas in the foreseeable future. Furthermore, expectations include a progressive reduction in health disparities and inequalities between these two geographical settings.

### Limitations

This study is subject to several limitations. Firstly, the adoption of a cross-sectional research design precludes the inference of causal relationships between digital literacy, health literacy, and digital health behaviors from the study findings. Future investigations would benefit from incorporating randomized controlled trials to provide a more nuanced understanding of the causal interplay among these three factors. Secondly, the generalizability of the study results to other countries and regions is constrained by data acquisition challenges and geographical limitations. In the future, our goal is to expand the sample size to include a broader national and global range, striving to derive research conclusions that are universally applicable. Lastly, both digital literacy and health literacy were gauged through self-reports, introducing the possibility of reporting bias. Future research endeavors could enhance methodological rigor by employing more objective measurement approaches, such as the assessment of digital literacy and health literacy through practical tasks within a digital health environment. This would mitigate potential distortions arising from self-reported data, ensuring more accurate and reliable measurements.

## Conclusion

Notwithstanding these limitations, the present research makes noteworthy contributions. Primarily, it stands out as the inaugural study delving into the interplay between digital literacy, health literacy, and digital health behavior among rural residents. The findings pave the way for a novel approach to enhancing the health status of rural residents in the future—by first elevating their digital literacy and health literacy. Secondly, the regression model adeptly controls for a plethora of confounding factors, bolstering the robustness of the results. Concurrently, the conclusions undergo scrutiny through instrumental variable methods, robustness tests, and heterogeneity analyses, fortifying the study's overall robustness.

### Supplementary Information

**Supplementary Material 1.**

**Supplementary Material 2.**

## Data Availability

The original contributions presented in the study are included in the article/supplementary material, further inquiries can be directed to the corresponding author.
