# Supplementary material for: Associations between digital literacy, health literacy, and digital health behaviors among rural residents: evidence from Zhejiang, China
Source: Int J Equity Health. 2024 Apr 9;23:68. doi: 10.1186/s12939-024-02150-2 (PMC11003150; doi:10.1186/s12939-024-02150-2)
Supplement: Supplementary file 1 — Supplementary Material 1. [file 12939_2024_2150_MOESM1_ESM.docx]

**Appendix 1**

**农村居民数字素养测度体系（Digital Literacy Measurement System for Rural Residents）**

| **维度**  **Dimensions** | **题项**  **Items** | **赋值**  **Value** |
| --- | --- | --- |
| General Digital Literacy  数字通用素养 | Can you use the general functions of a smartphone?  是否会使用智能手机的一般功能？ | Yes=1; No=0  是=1; 否=0 |
|  | Can you operate basic applications on a computer?  是否会操作电脑的简单应用？ | Yes=1; No=0  是=1; 否=0 |
| Digital Social Literacy  数字社交素养 | Can you use social software (WeChat, QQ, etc.) to chat with family and friends?  是否会使用社交软件（微信、QQ等）与家人、朋友聊天？ | Yes=1; No=0  是=1; 否=0 |
|  | Can you use social software (WeChat, QQ, etc.) to share interesting information links with family and friends?  是否会使用社交软件（微信、QQ等）向家人、朋友分享有趣的信息？ | Yes=1; No=0  是=1; 否=0 |
| Digital Search Literacy  数字搜寻素养 | Can you retrieve the data or information you want through the internet?  是否可以通过网络检索自己想要获得的数据或信息？ | Yes=1; No=0  是=1; 否=0 |
|  | Can you use different search platforms (browsers, Douyin, Zhihu, etc.) for information retrieval?  是否可以使用不同检索平台（浏览器、抖音、知乎等）进行信息搜寻？ | Yes=1; No=0  是=1; 否=0 |
|  | Can you save or bookmark the retrieved content using your mobile phone?  是否可以使用手机对检索到的内容进行保存或收藏？ | Yes=1; No=0  是=1; 否=0 |
| Digital Creative Literacy  数字创意素养 | Can you create short videos related to life and work using your mobile phone?  是否可以通过手机制作与生活和工作相关的短视频？ | Yes=1; No=0  是=1; 否=0 |
|  | Can you express your opinions and views online?  是否可以在网络上发表自己的意见和观点？ | Yes=1; No=0  是=1; 否=0 |
|  | Can you engage in online live streaming using your mobile phone or computer?  是否可以利用手机或电脑进行网络直播？ | Yes=1; No=0  是=1; 否=0 |
| Digital Safety Literacy  数字安全素养 | When using online social tools like WeChat, do you consider issues related to account and password security?  使用微信等线上社交工具时，是否考虑账号、密码等信息安全问题？ | Yes=1; No=0  是=1; 否=0 |
|  | Can you discern online advertisements or fraudulent information?  是否能够判断网络上的广告或欺诈信息？ | Yes=1; No=0  是=1; 否=0 |
|  | Can you change passwords for applications such as Alipay and online banking by yourself without needing assistance from others?  你自己是否会修改支付宝、网上银行等应用程序的密码？ | Yes=1; No=0  是=1; 否=0 |
