# Supplementary material for: Associations between digital literacy, health literacy, and digital health behaviors among rural residents: evidence from Zhejiang, China
Source: Int J Equity Health. 2024 Apr 9;23:68. doi: 10.1186/s12939-024-02150-2 (PMC11003150; doi:10.1186/s12939-024-02150-2)
Supplement: Supplementary file 2 — Supplementary Material 2. [file 12939_2024_2150_MOESM2_ESM.docx]

**Appendix 2**

**农村居民健康素养测度体系（Health Literacy Measurement System for Rural Residents）**

| **维度**  **Dimensions** | **题项**  **Items** | **赋值**  **Value** |
| --- | --- | --- |
| 健康理念  Health Philosophy | 您是否赞同“保健食品不能替代药物”这种说法？ A.赞同 B.不赞同  Do you agree with the statement "Health supplements cannot replace medicine"? A. Agree B. Disagree | A=1; B=0 |
|  | 您是否赞同“打针比吃药好得快，生病了就应该尽量打针”这种说法？ A.赞同 B.不赞同  Do you agree with the statement "Getting injections is faster and better than taking medicine; one should try to get injections when sick"? A. Agree B. Disagree | A=0; B=1 |
|  | 您是否赞同“每年应该做1次健康体检”这种说法？ A.赞同 B.不赞同  Do you agree with the statement "One should have a health checkup once a year"? A. Agree B. Disagree | A=1; B=0 |
| 健康知识  Health Knowledge | 您认为以下哪组血压属于正常血压值？ A.140/95毫米汞柱 B.150/100毫米汞柱 C.120/80毫米汞柱  Which blood pressure reading do you consider to be within the normal range? A. 140/95 mmHg B. 150/100 mmHg C. 120/80 mmHg | A=1; B=0; C=2 |
|  | 您认为什么时候是流感的高发期？ A.春夏季节 B.秋冬季节 C.冬春季节  When do you think is the peak season for influenza? A. Spring and summer B. Autumn and winter C. Winter and spring | A=0; B=2; C=1 |
|  | 您认为奶类主要可以提供哪些营养成分？ A.蛋白质、脂肪 B.钙、磷、钾等矿物质 C.脂肪  What nutrients do you think dairy products primarily provide? A. Protein, fat B. Calcium, phosphorus, potassium C. Fat | A=2; B=0; C=1 |
|  | 您认为长期大量饮酒对人体哪个脏器损害最严重? A.心脏 B.肝脏 C.肾脏 D.不知道  Which organ do you think is most severely damaged by long-term heavy drinking? A. Heart B. Liver C. Kidneys D. Don't know | A=1;B=2;C=1;D=0 |
|  | 您认为长期使用铝制品作为食品容器会引发下列哪种疾病？ A.老年痴呆症 B.甲状腺肿大 C.肠胃疾病  Which disease do you think may be triggered by the long-term use of aluminum products as food containers? A. Alzheimer's disease B. Thyroid enlargement C. Gastrointestinal diseases | A=2; B=0; C=1 |
| 健康技能  Health Skills | 您在阅读关于健康话题的说明书或其他材料时，是否需要别人的帮助？ A.不需要 B.有时需要 C.经常需要 D.总是需要  When reading instructions or other materials about health topics, do you need assistance from others? A. Not needed B. Sometimes needed C. Often needed D. Always needed | A=3;B=2;C=1;D=0 |
|  | 发生煤气中毒后，救护者首先应该怎样处理煤气中毒的人?（多选） A.迅速打开门窗 B.给病人盖上大衣或毛毯、棉被 C. 对未昏迷者，可以喂些浓茶、鲜萝卜汁、绿豆汤 D. 不知道  After a gas poisoning incident, what should the rescuer do first to treat the person affected by gas poisoning? (Multiple choices) A. Quickly open doors and windows B. Cover the patient with a coat, blanket, or quilt C. For those who are not unconscious, you can feed them strong tea, fresh carrot juice, mung bean soup D. Don't know | ABC=3; AB or AC or BC=2; A or B or C=1; D=0 |
